# Sarcopenia as Manifested by L3SMI Is Associated with Increased Long-Term Mortality amongst Internal Medicine Patients—A Prospective Cohort Study

**DOI:** 10.3390/jcm11123500

**Published:** 2022-06-17

**Authors:** Doron Portal, Guy Melamed, Gad Segal, Edward Itelman

**Affiliations:** 1 Sackler School of Medicine, Tel Aviv University, Ramat-Aviv, Tel Aviv 6997801, Israel; dorongn@gmail.com (D.P.); guy121233@gmail.com (G.M.); 2 Sheba Medical Center, Tel Aviv 6997801, Israel; gad.segal@sheba.health.gov.il

**Keywords:** sarcopenia, frailty, L3SMI, mortality, internal medicine

## Abstract

**Background:** Sarcopenia and Frailty are syndromes that affect the clinical outcomes of patients suffering from a wide range of diseases. The use of Computed Tomography (CT) is well established for Sarcopenia evaluation via estimation of the Skeletal Muscle Index (SMI) at the level of the third lumbar vertebra (L3SMI). Nevertheless, the association of more readily available biomarkers of Sarcopenia and clinical outcomes is desired. Recent studies have associated low Alanine amino-transferase ALT (SGPT) levels with Sarcopenia and frailty. The current study aimed to establish the association between low L3SMI and the aforementioned indices of Sarcopenia, frailty and poor clinical outcomes. **Methods:** A cohort study of patients admitted to the internal medicine department at a tertiary medical center. Sarcopenia was determined as L3SMI, lower than 53 cm^2^/m^2^ in men and 41 cm^2^/m^2^ in women. Clinical and mortality data was collected from the medical record. **Results:** Of the 187 patients recruited (mean age 70.4 ± 9.2, 59% males), 116 (62%) had Sarcopenia, based on L3SMI values. Sarcopenic patients were older, predominantly male, had lower BMI, lower mid-arm muscle circumference (MAMC) and low ALT values upon admission. L3SMI values significantly correlated with age and MAMC among males (R = −0.38, *p* < 0.001, R = 0.35, *p* < 0.001, respectively). Sarcopenia was associated with higher, one-year mortality (HR = 2.60, 95% CI 1.06–6.37, *p* = 0.036) and shorter all-time survival (HR = 2.91, 95% CI 1.35–6.29, *p* = 0.007). The association with all-time survival remained after adjusting for age and sex (HR = 2.38, 95% CI 1.07–5.29, *p* = 0.034). **Conclusion**: As defined by low L3SMI value, Sarcopenia is a poor prognostic factor for the general internal ward patient population. As part of personalized medicine, physicians may benefit from measuring L3SMI value, as indicated by commonly performed CT scans, to objectively assess their patient’s risk of suffering from Sarcopenia and frailty-associated complications.

## 1. Introduction

While life expectancy increases rapidly worldwide, the prevalence of Sarcopenia continues to rise. Optimal diagnosis and care for patients with Sarcopenia are essential more than ever since this common medical condition is associated with high personal, social, and economic burdens. It has been established that the presence of Sarcopenia is related to the development of several diseases and is associated with increased risk of hospitalization, increased cost and duration of hospital stay [1] and lower quality of life [2].

In 1989, Rosenberg proposed the term sarcopenia, derived from the Greek word “Sarx” for flesh and “Penia” for loss, to describe this age-related decrease of muscle mass [3]. The clinical syndrome is characterized by progressive and generalized loss of skeletal muscle mass and strength, leading to the phenotype of frailty. Although the disease is primarily a condition of the elderly, its development may be derived from conditions that are not exclusively correlated with older people, such as patients undergoing hemodialysis, suffering from heart failure, or diagnosed with diabetes mellitus [4,5]. Frailty may be objectively evaluated using various scales and markers, including low Alanine amino-transferase ALT (SGPT), low albumin and FRAIL questionnaire scores, all of which have been shown to correlate with lower survival rates [6,7].

While pharmaceutical therapies might be beneficial for those who suffer from sarcopenia [8], currently, the mainstay of sarcopenia treatment is focused on resistance exercise to increase muscle mass [9]. Early diagnosis and constant follow-ups are critical for slowing disease progression.

Over the years several definitions for Sarcopenia have been developed [10]. The consensus requires functional impairment (for example, poor grip strength), in addition to low muscle mass. A few different methods for lean body mass measurements are available with varying levels of accuracy. These methods include air displacement plethysmography, bioelectrical impendence analysis, ultrasound, and computed tomography (CT) [2].

Sarcopenia has been proven to significantly affect a wide range of diseases and health conditions as a marker for a higher risk of prospective complications, particularly in patients with cancer [11]. The disease effect differs among populations, but overall, it has been associated with increased disabilities and mortality among patients who meet its criteria [12]. 

The tests and tools used for sarcopenia characterization in practice may depend upon the patient’s physical capabilities and the healthcare test settings and resources [13]. CT is considered the gold standard modality for Sarcopenia evaluation due to its accuracy in body composition measurement and its availability in the clinical setting [14]. Research has shown that three-dimensional muscle mass at the level of L3 vertebra on two-dimensional planner sections of the CT scan enables a good estimation of whole-body muscle mass. The muscle area at the level of the L3 vertebra, divided by the square of the patient’s height, is accepted as a surrogate marker of Sarcopenia, also known as L3 Skeletal Muscle Index (L3SMI) [9,15].

This study aim was to test the correlation between Sarcopenia, diagnosed by L3SMI criteria, and long-term mortality among patients admitted to the internal medicine ward. 

## 2. Methods

### 2.1. Study Population

This was a prospective cohort study of all adult patients aged over 49 hospitalized in the internal medical ward at a tertiary medical center, between November 2018 and October 2019 who completed a CT scan eligible for evaluation of the L3SMI. Patients with poor prognosis were excluded: advanced (stage IV) malignancy, chronic obstructive pulmonary disease GOLD stage IV, progressive dementia, congestive heart failure (CHF) NYHA stage III and IV, history of a major stroke, end-stage cirrhosis, diabetic foot or severe peripheral vascular disease, bed-ridden patients, patients requiring mechanical ventilation and patients hospitalized during the last 30 days before the index admission. The Institutional Review Board of the Sheba Medical Centre approved this study (protocol code: 8415-21-SMC), and all patients signed informed consent forms prior to participating in the study.

### 2.2. Variable Definition

The principal investigated predictor, L3SMI, was evaluated based on patients’ CT scans, performed within six months prior and three months post-admission. If multiple scans were available in this period, the closest scan to the index hospitalization was chosen. Skeletal muscle volume was calculated using AW Server software (General Electric Healthcare Inc., Chicago, IL, USA) and was performed by two investigators (interobserver correlation R = 0.99, *p* < 0.001). The muscle area was corrected for the slice thickness and divided by the patient squared height to achieve the skeletal muscle index. Patients whose scans demonstrated values under 41 cm^2^/m^2^ for females and 53 cm^2^/m^2^ for males were considered as diagnosed with Sarcopenia. BMI specific cutoffs were examined, where males with BMI under 25 and SMI under 43 cm^2^/m^2^ were considered sarcopenic [16].

Upon admission, patients were assessed for frailty using the Frail questionnaire, scoring five items (fatigue, resistance, ambulation, illnesses, and loss of weight). FRAIL scores represent frail (3–5), pre-frail (1–2), and robust (0) health statuses. In this study, we used frail as a categorical variable—frail (3–5) and non-frail (0–2) [6]. Mid-Arm Muscle Circumference (MAMC) was calculated from the Mid-Arm Circumference (MAC) and Triceps Skin Fold (TSF) measurements upon admission, using the equation MAMC = MAC-(3.14XTSF). Measurements were performed thrice, and the mean value was used. All participants were interviewed and measured by the same single researcher.

Upon admission, additional frailty variables measured were Norton and Morse scales [17,18], and ALT level. We used an ALT level of below 12IU to represent low ALT in the cohort [5]. Laboratory tests values upon admission (including albumin, hemoglobin, creatinine, glucose, C-reactive protein, white blood cells, and platelets), recorded comorbidities, cause of admission and demographic variables (including marital status, age, sex, and BMI) were included as well.

The primary outcome was all-cause mortality and death within one year of admission. Mortality data was received from the Israeli national population registry.

### 2.3. Statistical Analysis

Categorical variables were described as numbers and percentages. Continuous variables were examined for their distribution using histogram and Q–Q plot and described as mean and standard deviation for normally distributed variables or median and IQR for other variables. Association between L3SMI and continuous variables was evaluated using Pearson or Spearman correlation coefficient. Continuous variables were compared between L3SMI categories using independent sample *t*-test or Mann–Whitney tests. Categorical variables were compared using chi-square or Fisher exact test. Length of follow-up was described using the reverse censoring method. Kaplan–Meier curve was used to describe survival, and the log-rank test was applied to compare L3SMI categories. All variables were explored for an association with all-cause mortality using univariate Cox regression. In order to maintain the rule of ten events per variable, the multivariable analysis included only three variables—L3SMI (Low vs. normal) and two common confounders (age and sex). All statistical tests were two-sided, and a *p*-value below 0.05 was considered significant. All statistical analyses were performed using SPSS (IBM Corp. Released 2017. IBM SPSS Statistics for Windows, Version 25.0. Armonk, NY, USA).

## 3. Results

### 3.1. Study Population 

Out of the 979 eligible admitted patients, 187 had a relevant CT scan and were included in this study. The mean age was 70.4 ± 9.2 and 111 (59%) were males. The median follow-up time was 29.4 months (IQR 25.1–31.9 months). Based on their CT scan, 116 patients (62%) were sarcopenic as defined by low L3SMI value (Table 1).

Sarcopenic patients were older (72 ± 8.9, vs. 67.7 ± 9.1, *p* = 0.002), predominantly male (68.1% vs. 45.1%, *p* = 0.002) and had lower BMI than their non-sarcopenic counterparts (median BMI 25 (IQR: 22.4–27.5) vs. 27.7 (IQR: 25–32), *p* < 0.001). Rates of pre-existing comorbidities did not differ between the two groups, as were most laboratory values upon admission, excluding slightly lower White Blood Cell count among sarcopenic patients (Table 1).

### 3.2. Frailty Measurements

Frailty measurements were found to be significantly different between the two groups. Sarcopenic patients were more likely to have low ALT levels defined as less than 12 International units at admission—26.7% vs. 10.1%, *p* = 0.007 and lower mid-arm muscle circumference (MAMC) (median 26.2 cm (IQR: 24.6–28.9) vs. 28.1 (IQR: 26.2–30.7), *p* < 0.001). The frequency of a total score of above three in the Frail questionnaire did not differ between the two groups (64.7% vs. 56.3%, *p* = 0.257). 

When examining the correlation between L3SMI values and other frailty and sarcopenia measurements, among males, higher L3SMI values correlated with younger age (R = −0.38, *p* < 0.001) and high MAMC (R = 0.35, *p* < 0.001). The same correlation was absent among females. Interestingly, both Norton and Morse scales correlated significantly with L3SMI value, MAMC, ALT, and FRAIL questionnaire among males, but not quite among females (Norton: R = 0.25, *p* = 0.007, R = 0.25, *p* = 0.008, R = 0.30, *p* = 0.002, R = −0.40, *p* < 0.001, and Morse: R = −0.27, *p* = 0.004, R = −0.19, *p* = 0.046, R = −0.23, *p* = 0.015, R = 0.35, *p* < 0.001, respectively, among males). Neither ALT value nor FRAIL questionnaire correlated with L3SMI in this cohort (R = −0.25, *p* = 0.791 and R = −0.149, *p* = 0.119, respectively, for males and R = −0.027, *p* = 0.821, R = −0.083, *p* = 0.478, respectively, for females). All correlations appear in Supplementary Table S1.

### 3.3. Long Term Survival

During the first year of follow-up, 30 patients died. Of them, a single patient died during the index hospitalization. An additional 12 patients died during the next two years of follow-up, marking a total of 42 (22.5%) deaths.

**Figure 1:**
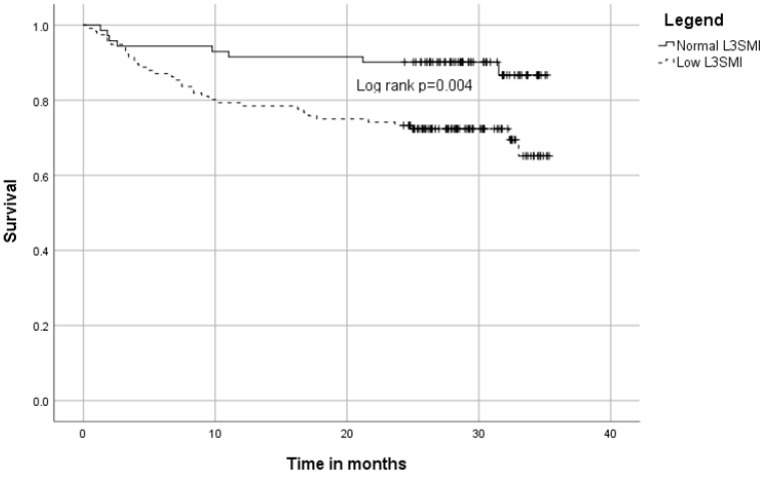
Kaplan–Meier plot of low L3SMI and mortality.

Low L3SMI was associated with lower one-year survival rates (HR = 2.60, 95% CI 1.06–6.37, *p* = 0.036). This was more prominent for all-time survival, in which sarcopenic patients were almost three times more likely to die than their non-sarcopenic counterparts (HR = 2.91, 95% CI 1.35–6.29, *p* = 0.007). As demonstrated by the survival plot, the mortality of both groups is similar in the first three months post-admission (Figure 1). Results did not differ when using BMI-specific cutoff values (Supplementary Table S2).

Additional predictors of long-term mortality in our cohort were hemoglobin levels, albumin levels, age, pre-existing diabetes mellitus, and high Norton and Morse scales scores. Congestive heart failure and chronic renal failure were predictors of one-year survival but not all-time survival (Table 2).

Adjusted to age and sex, low L3SMI was associated with all-time mortality (HR = 2.38, 95% CI 1.07–5.29, *p* = 0.034) but not one-year mortality (HR = 2.07, 95% CI 0.81–5.27, *p* = 0.127) (Table 3).

## 4. Discussion

Since Rosenberg proposed the term sarcopenia in 1989 [3], much has been studied and established about the syndrome and how a precise evaluation of the disease’s stage is significant for morbidity and mortality. The rise in the prevalence of the syndrome, following the increase in life expectancy, in addition to the lack of proper therapeutic means to treat Sarcopenia and frailty [8], emphasizes the need for accurate and accessible standards for diagnosis and characterization of the two medical conditions [1]. Sarcopenia and frailty are found not only among the elderly but rather also in younger patients undergoing various diseases [5]. Therefore, the primary goal of the majority of recent research has been developing tools for early detection of patients with Sarcopenia in clinical practice to prevent deterioration of one’s condition and age-associated disability [19]. CT images of a specific lumbar vertebral landmark (L3) are correlated significantly with whole-body muscle evaluation. Currently, CT is considered the gold standard for Sarcopenia evaluation due to its accuracy in body composition measurement and its availability in the clinical setting [20,21]. As a result, this imaging method is used to detect low muscle mass, even in patients with normal or high body weights, and it can also predict prognosis in various diseases [22,23,24]. L3SMI is not limited to patients with cancer; for example, this parameter has been used as a predictor of mortality and other outcomes in the intensive care unit [25]. Cruz-Jentoft et al. [1], as part of the European Working Group on Sarcopenia in Older People (EWGSOP), stated that with ever-increasing needs to quantify muscle mass and detect Sarcopenia in early stages, high-resolution imaging is expected to be used more widely in the future. Initially in research and studies, and ultimately in clinical practice. Studies such as this one could help validate L3SMI as a leading imaging-based method of sarcopenia evaluation in a wide range of patients and diseases. The application of the L3SMI measurement should join other biomarkers of Sarcopenia (e.g., ALT or DEXA whenever available) and phenotypic functional tests (e.g., FRAIL questionaries). 

In this study, we have shown that a low L3SMI value is associated with mortality. The effect remained for long-term mortality after adjusting for age and sex. A similar survival rate for sarcopenic and non-sarcopenic patients in the first three months after the index hospitalization has been demonstrated. This finding can be expected; hence Sarcopenia is a progressive process associated with advancement in the underlying disease, making it a better marker for long-term mortality than short-term. While L3SMI was validated as a prognostic tool for specific patient populations, this prospective cohort study is the first to examine it in the general inpatient population that inhabits the internal ward. The prospective nature also allowed us to test multiple frailty assessment tools and correlate them with Sarcopenia and other prognostic measures. 

Sarcopenia and frailty were long considered geriatric syndromes, associated with increased risk of falls, frequent hospitalizations, and death. In recent years, it has been widely accepted that these two, “twin” syndromes should be applied to earlier ages—in accord with the realization that the biologic, rather than the chronologic age of patients is the more important factor. Customizing the intensity of applying diagnostic procedures and therapeutic interventions according to the biological age of patients should be considered as personalized medicine. Fine-tuning of therapy according to the existence of Sarcopenia and frailty should be conducted for the benefit of the patients and society altogether.

This study has a few limitations. Only patients who had available CT scans were included in our analysis; this might create a selection bias. Data regarding myopathy risk due to pre-existing chronic disease, or chronic medication use (i.e., corticosteroids) were not available, and thus were not evaluated in this study. Another limitation is the small number of events limiting the extent of our multivariate models. To eliminate this constraint, future studies should aspire to have larger prospective cohorts with standardized CT scans.

## 5. Conclusions

In the current study, we show that L3SMI could join the sarcopenia-frailty evaluation “kit” of physicians. No longer should eyeballing the patients be considered appropriate. Before aggressive diagnostics (e.g., biopsies and other invasive procedures) and intensive therapeutic interventions (e.g., chemotherapy and potentially curative surgery) a thorough evaluation of Sarcopenia and frailty should be the rule. For many patients who went through CT tests in the last six months, appreciation of the L3SMI should be considered.

## Figures and Tables

**Table 1:** Patients’ characteristics.

Characteristics	All Patients	Sarcopenic (*n* = 116)	Non-Sarcopenic (*n* = 71)	*p* Value
Age (years), mean ± SD	70.4 ± 9.2	72.0 ± 8.9	67.7 ± 9.1	**0.002**
Male, *n* (%)	111 (59.4)	79 (68.1)	32 (45.1)	**0.002**
BMI, median (IQR)	25.8 (23.1–28.7)	25.0 (22.4–27.5)	27.7 (25.0–32.0)	**<0.001**
Comorbidity, *n* (%)				
CHF	17 (9.1)	8 (6.9)	9 (52.9)	0.182
COPD	38 (20.3)	26 (22.4)	12 (16.9)	0.363
CRF	32 (17.1)	23 (19.8)	9 (12.7)	0.208
DM	82 (43.9)	52 (44.8)	30 (42.3)	0.731
HTN	108 (57.8)	66 (56.9)	42 (59.2)	0.762
IHD	55 (29.4)	35 (30.2)	20 (28.2)	0.770
Solid malignancy	33 (17.6)	22 (19.0)	11 (15.5)	0.545
Cardiac cause of admission, *n* (%)	30 (16)	19 (16.4)	11 (15.5)	0.873
Infectious cause of admission, *n* (%)	24 (12.8)	13 (11.2)	11 (15.5)	0.395
MAMC, median (IQR)	26.9 (25.0–29.5)	26.2 (24.6–28.9)	28.1 (26.2–30.7)	**<0.001**
ALT below 12	38 (20.5)	31 (26.7)	7 (10.1)	**0.007**
FRAIL score 3+	115 (61.5)	75 (64.7)	40 (56.3)	0.257

SD: Standard Deviation; BMI: Body Mass Index; IQR: Interquartile range; CHF: Congestive Heart Failure; COPD: Chronic Obstructive Pulmonary Disease; CRF: Chronic Renal Failure; DM: Diabetes Mellitus; HTN: Hypertension; IHD: Ischemic Heart Disease; MAMC: Middle-Arm Muscle Circumference; ALT: Alanine Aminotransferase. Bold indicates statistical significance.

**Table 2:** Association with survival—Univariate analysis.

	One Year Survival		All Time Survival	
	HR (95% CI)	*p* Value	HR (95% CI)	*p* Value
Low L3SMI	2.60 (1.06–6.37)	**0.036**	2.91 (1.35–6.29)	**0.007**
Age	1.06 (1.02–1.11)	**0.008**	1.06 (1.02–1.10)	**0.003**
Female sex	0.72 (0.34–1.54)	0.401	0.70 (0.37–1.33)	0.278
BMI	0.98 (0.91–1.06)	0.688	0.97 (0.91–1.08)	0.382
Comorbidity				
IHD	1.05 (0.48–2.30)	0.901	0.76 (0.37–1.54)	0.441
CHF	3.05 (1.25–7.47)	**0.015**	2.06 (0.87–4.89)	0.102
HTN	1.11 (0.54–2.31)	0.775	0.98 (0.53–1.80)	0.934
CRF	2.20 (1.01–4.80)	**0.048**	1.88 (0.95–3.75)	0.072
DM	1.75 (0.85–3.61)	0.127	1.99 (1.08–3.69)	**0.028**
COPD	1.77 (0.81–3.86)	0.152	1.75 (0.90–3.43)	0.102
Solid malignancy	0.90 (0.34–2.34)	0.821	0.95 (0.42–2.14)	0.898
Marital Status		0.099		0.107
Married	REF			
Divorced	1.05 (0.35–3.13)		0.90 (0.33–2.26)	
Widowed	2.74 (1.22–6.15)		2.31 (1.14–4.69)	
Other	--		--	
Albumin	0.29 (0.15–0.57)	**<0.001**	0.41 (0.23–0.72)	**0.002**
Hemoglobin	0.85 (0.73–0.98)	**0.030**	0.80 (0.70–0.91)	**<0.001**
MAMC	0.96 (0.87–1.07)	0.503	0.94 (0.86–1.03)	0.182
ALT above 12	0.69 (0.31–1.55)	0.370	0.67 (0.34–1.34)	0.255
FRAIL score 3+	1.79 (0.80–4.01)	0.160	1.24 (0.66–2.33)	0.508

L3SMI: Lumbar Vertebrae 3 Skeletal Muscle Index, SD: Standard Deviation, BMI: Body Mass Index IQR: Interquartile range, CHF: Congestive Heart Failure, COPD: Chronic Obstructive Pulmonary Disease, CRF: Chronic Renal Failure, DM: Diabetes Mellitus, HTN: Hypertension, IHD: Ischemic Heart Disease, MAMC: Middle-Arm Muscle Circumference, ALT: Alanine Aminotransferase. Bold indicates statistical significance.

**Table 3:** Association with survival—Multivariate analysis.

	One Year Survival		All Time Survival	
	HR (95% CI)	*p* Value	HR (95% CI)	*p* Value
Low L3SMI	2.07 (0.81–5.27)	0.127	2.38 (1.07–5.29)	**0.034**
Age	1.05 (1.01–1.10)	**0.026**	1.05 (1.01–1.09)	**0.016**
Female sex	0.91 (0.42–1.99)	0.814	0.91 (0.47–1.76)	0.777

HR: Hazard Ratio; L3SMI: Lumbar Vertebrae 3 Skeletal Muscle Index. Bold indicates statistical significance.

## Data Availability

Not applicable.

## Supplementary Materials

The following supporting information can be downloaded at: https://www.mdpi.com/article/10.3390/jcm11123500/s1, Table S1: Correlation between L3SMI and different frailty measurements, per sex category. Table S2: BMI Specific cut-off values for Skeletal Muscle Index.
