# Supplementary material for: Sarcopenia as Manifested by L3SMI Is Associated with Increased Long-Term Mortality amongst Internal Medicine Patients—A Prospective Cohort Study
Source: J Clin Med. 2022 Jun 17;11(12):3500. doi: 10.3390/jcm11123500 (PMC9224962; doi:10.3390/jcm11123500)
Supplement: Supplementary file 1 [file jcm-11-03500-s001.zip › jcm-1724956-supplementary.pdf]

Supplementary Table S1- Correlation between L3SMI and different frailty measurements, per sex category.

A. Sex=Male

Pearson Correlations

		L3SMI	Age	Hemoglobin	Albumin
L3SMI	Pearson Correlation	1	-.382**	.142	.172
	Sig. (2-tailed)		.000	.137	.073
	N	111	111	111	110
Age	Pearson Correlation	-.382**	1	-.248**	-.011
	Sig. (2-tailed)	.000		.009	.913
	N	111	111	111	110
Hemoglobin	Pearson Correlation	.142	-.248**	1	.503**
	Sig. (2-tailed)	.137	.009		.000
	N	111	111	111	110
Albumin	Pearson Correlation	.172	-.011	.503**	1
	Sig. (2-tailed)	.073	.913	.000	
	N	110	110	110	110

**. Correlation is significant at the 0.01 level (2-tailed).

			L3SMI	MAMC	ALT	Norton admission	Morse admission	FRAIL SCORE
**Spearman's Rho**	L3SMI	Correlation Coefficient	1.000	.351**	-.025	.254**	-.268**	-.149
		Sig. (2-tailed)	.	.000	.791	.007	.004	.119
		N	111	111	111	111	111	111
	MAMC	Correlation Coefficient	.351**	1.000	.071	.250**	-.190*	-.157
		Sig. (2-tailed)	.000	.	.457	.008	.046	.100
		N	111	111	111	111	111	111
	ALT	Correlation Coefficient	-.025	.071	1.000	.298**	-.231*	-.085
		Sig. (2-tailed)	.791	.457	.	.002	.015	.374
		N	111	111	111	111	111	111
		N	17	17	17	17	17	17

	Norton admission	Correlation Coefficient	.254**	.250**	.298**	1.000	-.605**	-.399**
		Sig. (2-tailed)	.007	.008	.002	.	.000	.000
		N	111	111	111	111	111	111
	Morse admission	Correlation Coefficient	-.268**	-.190*	-.231*	-.605**	1.000	.347**
		Sig. (2-tailed)	.004	.046	.015	.000	.	.000
		N	111	111	111	111	111	111
	FRAIL SCORE	Correlation Coefficient	-.149	-.157	-.085	-.399**	.347**	1.000
		Sig. (2-tailed)	.119	.100	.374	.000	.000	.
		N	111	111	111	111	111	111

B. Sex=Female

Pearson Correlations

		L3SMI	Age	Hemoglobin	Albumin
L3SMI	Pearson Correlation	1	-.118	-.072	.058
	Sig. (2-tailed)		.309	.534	.632
	N	76	76	76	71
Age	Pearson Correlation	-.118	1	-.318**	-.180
	Sig. (2-tailed)	.309		.005	.134
	N	76	76	76	71
Hemoglobin	Pearson Correlation	-.072	-.318**	1	.335**
	Sig. (2-tailed)	.534	.005		.004
	N	76	76	76	71
Albumin	Pearson Correlation	.058	-.180	.335**	1
	Sig. (2-tailed)	.632	.134	.004	
	N	71	71	71	71

**. Correlation is significant at the 0.01 level (2-tailed).

a. Gender = 1

			L3SMI	MAMC	ALT	Norton admission	Morse admission	FRAIL SCORE
Spearman's rho	L3SMI	Correlation Coefficient	1.000	.224	-.027	-.045	.093	-.083
		Sig. (2-tailed)	.	.051	.821	.697	.425	.478
		N	76	76	74	76	76	76
	MAMC	Correlation Coefficient	.224	1.000	-.033	-.125	.166	.080
		Sig. (2-tailed)	.051	.	.780	.281	.151	.492
		N	76	76	74	76	76	76
		Sig. (2-tailed)	.020	.000	.546	.148	.093	.175
		N	75	75	73	75	75	75
	ALT	Correlation Coefficient	-.027	-.033	1.000	-.080	-.003	-.169
		Sig. (2-tailed)	.821	.780	.	.498	.981	.150
		N	74	74	74	74	74	74
	Norton admission	Correlation Coefficient	-.045	-.125	-.080	1.000	-.609**	-.157
		Sig. (2-tailed)	.697	.281	.498	.	.000	.175
		N	76	76	74	76	76	76
	Morse admission	Correlation Coefficient	.093	.166	-.003	-.609**	1.000	.117
		Sig. (2-tailed)	.425	.151	.981	.000	.	.313
		N	76	76	74	76	76	76
	FRAIL SCORE	Correlation Coefficient	-.083	.080	-.169	-.157	.117	1.000
		Sig. (2-tailed)	.478	.492	.150	.175	.313	.
		N	76	76	74	76	76	76

Supplementary Table S2- BMI Specific cut-off values for Skeletal Muscle Index

	**One Year Survival**		**All Time Survival**	
	HR (95%CI)	P value	HR (95%CI)	P value
BMI-Specific Low L3SMI	1.91 (0.89-4.07)	**0.096**	2.47 (1.26-4.82)	**0.008**
